# Convalescent Immunity to Guinea Pig Cytomegalovirus Induces Limited Cross Strain Protection against Re-Infection but High-Level Protection against Congenital Disease

**DOI:** 10.3390/ijms21175997

**Published:** 2020-08-20

**Authors:** K. Yeon Choi, Nadia S. El-Hamdi, Alistair McGregor

**Affiliations:** Department Microbial Pathogenesis & Immunology, College of Medicine, Texas A&M University, Bryan, TX 77807, USA; yeonchoi@tamu.edu (K.Y.C.); nselhamdi@exchange.tamu.edu (N.S.E.-H.)

**Keywords:** guinea pig, cytomegalovirus, vaccine, glycoproteins, neutralizing antibody, congenital CMV, pentamer complex, gO

## Abstract

The guinea pig is the only small animal model for congenital cytomegalovirus (cCMV) but requires guinea pig cytomegalovirus (GPCMV). Current GPCMV research utilizes prototype strain 22122, which limits the translational impact of GPCMV as numerous human CMV strains exist and cCMV is possible in the setting of re-infection. A novel strain of GPCMV (TAMYC) exhibited differences to 22122 in various glycoproteins with GP74 (gO homolog) the most variable (25% difference). Antibody ELISAs for TAMYC-convalescent animals evoked similar immune response to viral glycoprotein complexes (gB, gH/gL, gM/gN, pentamer) and cell-mediated response to pp65 homolog (GP83). Convalescent sera from TAMYC-infected animals neutralized GPCMV infection on fibroblasts but was less effective on epithelial cells. TAMYC-convalescent animals were not protected from dissemination of heterogenous virus challenge (22122). However, in a cCMV protection study, TAMYC-convalescent animals challenged mid-pregnancy (22122) exhibited high-level protection against cCMV compared to seronegative animals with pup transmission reduced from 80% (control) to 12%. Overall, pre-existing immunity in guinea pigs provides limited ability to prevent GPCMV re-infection by a different viral strain but provides a high level of protection against cCMV in heterogenous strain challenge. This level of cross protection against cCMV should be a prerequisite of any CMV vaccine.

## 1. Introduction

Human cytomegalovirus (HCMV), a betaherpesvirus, is a leading cause of congenital disease in newborns [1]. Congenital CMV (cCMV) can lead to serious symptomatic disease including cognitive and vision impairment as well as sensorineural hearing loss (SNHL) in newborns [1,2,3]. Globally, congenital HCMV occurs in approximately 1–5% of live births including areas with high seropositivity [4]. Alarmingly, cCMV can occur in women convalescent for the virus which may result from infection by a new strain and/or impaired immunity against the virus [4]. Consequently, cCMV can occur in mothers both seropositive and seronegative prior to pregnancy [5]. Ideally, a vaccine against cCMV should provide protection at the level higher than natural convalescent immunity. The viral gB glycoprotein which is essential for virus entry into all cell types, and an immunodominant neutralizing antibody target, remains a significant focus in various vaccine approaches. [6]. In clinical trials, a subunit gB vaccine attains at best about 50% efficacy against maternal infection [7]. Consequently, vaccine strategies that include the viral pentamer complex (PC), which is important for infection of non-fibroblast cells are also being evaluated [8]. Although the correlates of protection against cCMV are poorly defined, it is generally thought that neutralizing antibodies to viral glycoprotein complexes significantly contribute to protection, but immunity can also be enhanced by response to T cell target antigens (e.g., pp65) in convalescent individuals [9].

Intervention strategies against cCMV should ideally be investigated in a preclinical animal model. Species specificity of HCMV makes direct study of infection in animal models untenable. Species-specific animal CMV crosses the placenta in rhesus macaque (rhesus cytomegalovirus virus, RhCMV) and guinea pig (guinea pig cytomegalovirus, GPCMV) [10,11]. No CMV vaccines have been evaluated to date in a cCMV non-human primate (NHP) model. The guinea pig is the only small animal model for cCMV and the focus of this paper. Unlike mouse, the guinea pig and human placentas are hemomonochorial, containing a homogenous layer of trophoblast cells separating maternal and fetal circulation [12,13]. Congenitally infected newborn pups can have similar disease symptoms as humans, e.g., SNHL [14]. Therefore, the guinea pig is potentially well suited for evaluation of strategies against cCMV.

GPCMV encodes functional viral glycoprotein complexes to HCMV (gB, gH/gL/gO, gM/gN), which are important for virus cell entry [15,16,17]. Unlike murine cytomegalovirus (MCMV), GPCMV also encodes a pentamer complex (PC) found in clinical HCMV strains. The PC is a gH/gL based complex and in HCMV consists of gH/gL/UL128/UL130/UL131 viral proteins. The GPCMV homolog PC consists of gH/gL/GP129/GP131/GP133 [16]. Similar to HCMV, the GPCMV PC is essential for viral infection of non-fibroblast cell types [16] including placental cells leading to cCMV [16,18,19]. As with HCMV, the GPCMV viral glycoprotein complexes are important for neutralizing antibody targets [15,20,21,22]. GPCMV gB [21,23] is essential for infection of all cell types [15,16] and is the most extensively studied vaccine antigen against cCMV, either as a subunit vaccine [23], or expressed in recombinant vaccine vector delivery platforms [24,25,26,27]. These studies demonstrated that gB antibodies neutralized infection on fibroblast cells. However, in cCMV protection studies, the various gB vaccines attained approximately 50% efficacy [23,24,26,27]. Infection of non-fibroblast cells for both HCMV and GPCMV are dependent upon the PC for endocytic pathway cell entry and the process of PC-dependent entry is only partially defined. Expression of the viral cell receptor platelet-derived growth factor receptor alpha (PDGFRA) on fibroblasts enables HCMV and GPCMV cell entry by direct cell fusion independent of the PC but requires gH/gL/gO triplex and gB [28,29,30]. Despite the essential nature of gB for infection by HCMV and GPCMV, neutralizing antibodies to the PC might constitute a better vaccine target [31,32], especially since antibodies to the PC are more effective at virus neutralization on non-fibroblast cells including placental trophoblasts and amniotic sac membrane cells [8,19,25,29,33]. Additionally, GPCMV encodes homolog T cell target antigens to HCMV such as pp65 (GP83) and cell-mediated response to GP83 provides partial protection against cCMV [20,34,35].

Although various vaccine strategies against cCMV have been evaluated in the guinea pig, the most effective approach to date has been the use of a replication incompetent live viral strain, which is described as a Disabled Infectious Single Cycle (DISC) vaccine [20]. The GPCMV DISC vaccine incorporates various antibody and T cell target antigens and mimics natural infection but does not produce progeny virus in the host because of a lethal CMV capsid gene (*GP85*) mutation and requires a complementing cell line for virus production [20,29]. Protection against wild type virus challenge was significantly increased with high efficacy when this DISC vaccine incorporated the PC [29] compared to DISC (PC-) [20]. A PC+ DISC vaccine for HCMV is currently being evaluated in clinical trials [36]. All GPCMV animal studies to date were carried out in the context of vaccine protection against the same strain of virus (GPCMV strain 22122). An expectation of any successful HCMV vaccine is the ability to cross protect against multiple strains. Importantly, the antibody response to the PC has been shown to be important for broad spectrum neutralization of various HCMV clinical strains [32].

A current failing in GPCMV studies is that all vaccine candidates have been evaluated for protection against the prototype 22122 GPCMV strain (ATCC VR682) which was isolated in the 1950s and passaged multiple times on fibroblasts prior to being deposited with the ATCC [37]. Although cCMV challenge virus stocks for 22122 strain are generated serially in animals from salivary gland homogenate, potentially, strain 22122 might not typically represent viral strains present in animal colonies. Furthermore, the lack of available additional viral strains prevents the ability to evaluate immunity and vaccine cross strain protection against cCMV, which reduces the translational impact of GPCMV studies. Therefore, a new strain of GPCMV (designated TAMYC) was isolated from commercial animals. The encoded GPCMV glycoprotein genes and predicted proteins of TAMYC strain were compared to 22122 strain and TAMYC convalescent immunity determined for antibody and cell-mediated response. Additionally, the ability of convalescent immunity (TAMYC strain) in animals to cross-protect against heterogenous virus challenge (22122 strain) and dissemination to target organs was evaluated. Furthermore, the ability of TAMYC convalescent immunity to protect against cCMV infection by 22122 strain challenge was explored. Overall, results suggested a minimum threshold expectation for an effective vaccine strategy and cross strain protection against cCMV in this model.

## 2. Results

### 2.1. New Strain of GPCMV Has Hypervariable gO and Highly Epitrophic

Animals sourced from the same commercial holding room were identified as seropositive for GPCMV by anti-GPCMV ELISA. Seropositive animals were used to isolate GPCMV from salivary gland tissue. Initially, salivary gland tissue from individual animals was PCR screened for GPCMV DNA using primers to the viral polymerase subunit as described in materials and methods. Salivary gland tissue from one animal with high viral load was used to isolate the virus by co-culture of tissue homogenate with guinea pig renal epithelial cells [16].

**Figure 1:**
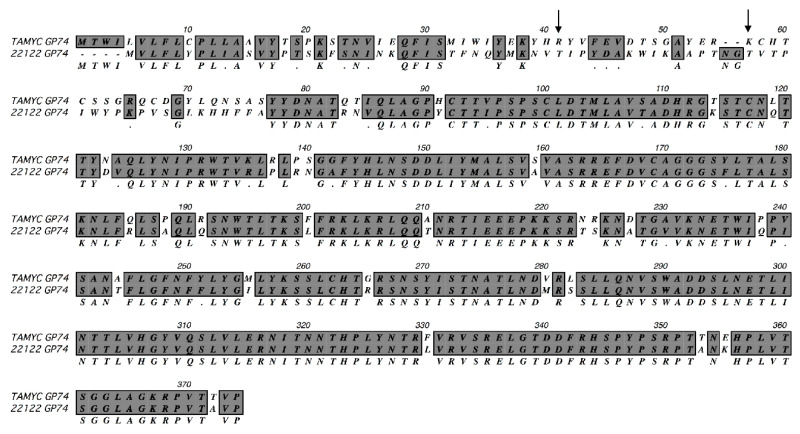
ClustalW protein alignment of TAMYC GP74 (gO). ClustalW alignment of predicted amino acid sequence of TAMYC GP74 (gO) compared to GP74 22122 strain (GenBank accession # AB592928). Sequence alignment was carried out using MacVector software. Gray background with consensus letter in the third row indicates amino acid sequence identity; gray background with black dot (.) in the third row indicates conservative amino acid substitution; unhighlighted with empty consensus row indicates mismatch. Arrow represents missing glycosylation sites (2) in the TAMYC strain. *N*-glycosylation consensus sequence = NXS/T.

Viral DNA was isolated from pass 1 virus stock from renal epithelial (REPI) cells in culture and used to PCR clone various virus glycoprotein genes: GP55 (gB); GP73 (gN); GP74 (gO); GP75 (gH); GP100 (gM); GP115 (gL); GP129 (HCMV UL128 homolog); GP131 (UL129 homolog); GP133 (UL131 homolog) as described in materials and methods using specific primer sets (Table S1) or previously described primers for GP100, GP115, GP129, GP131 and GP133 [15,16]. Three independent clones for each GPCMV glycoprotein gene were completely sequenced and consensus DNA and predicted amino acid sequence were determined. In HCMV, the predicted gO (UL74) glycoprotein amino acid sequence is the most diverse between various strains with 10–30% difference [38,39,40,41]. ClustalW protein alignment of the predicted GP74 (gO homolog) amino acid sequence [15] between GPCMV 22122 and newly isolated virus demonstrated significant differences in the coding sequence (75% identity), see Figure 1.

Consequently, the virus isolated from the commercial lab animal was provisionally considered to be a novel strain isolate and designated as TAMYC strain. The N-terminal half of the TAMYC strain GP74 protein encoded the majority of differences with 22122 strain GP74 and this is commonly observed between HCMV strains gO sequence [38,39,40,41]. Similar to HCMV gO, GP74 protein (22122 strain) is heavily glycosylated with 13 predicted sites [15]. In the TAMYC strain, the first two predicted N-glycosylation sites (N-X-T/A) are lost (amino acids 41 and 57) [15] (Figure 1) and the potential impact of this loss in this N-terminal domain glycosylation awaits further study but the second glycosylation site is conserved between HCMV and GPCMV gO [42]. Potentially modified glycosylation could impact on ability of gO to interact with PDGFRA cell receptor [30]. Sequence analysis of other viral glycoprotein genes and predicted proteins demonstrated additional differences between TAMYC and 22122 strains (Table 1, Figure S1).

The additional predicted individual glycoprotein amino acid sequence of TAMYC proteins exhibited varying levels of identity by ClustalW analysis with 22122 strain counterparts. The gH glycoprotein being the most variable (84% identity) after gO with all nine predicted N-glycosylation sites conserved (Figure S1) [15]. Other proteins had greater similarity between strains (88–99% amino acid identity), see Table 1, Figure S1A–F. Importantly, the TAMYC strain encoded unique components of the PC (GP129, GP131 and GP133) which were relatively strongly conserved with 22122 strain counterparts with minor changes at the nucleic acid and amino acid level (Table 1 and Figure S1D–F).

**Figure 2:**
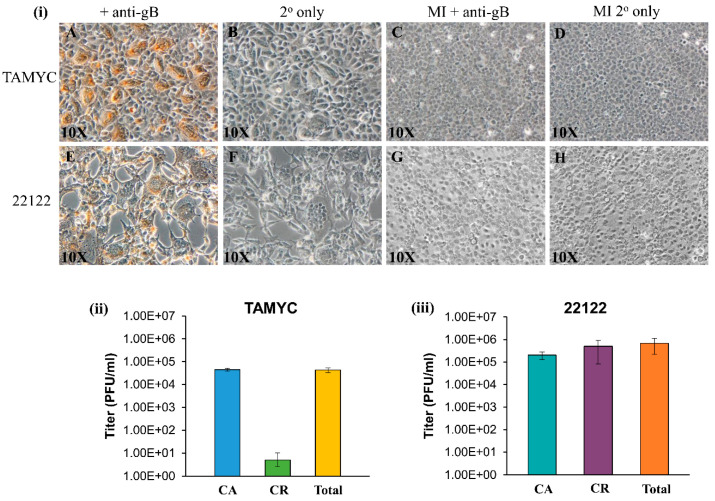
Comparison of TAMYC and 22122 GPCMV strain infection on renal epithelial (REPI) cells. (**i**) Viral gB protein staining of renal epithelial (REPI) cells infected with TAMYC strain (**A**–**D**) or 22122 strain (**E**–**H**) were carried out in six-well plates. Monolayers infected with TAMYC or 22122 strain GPCMV (moi 1 pfu/cell) (**A**,**B**,**E**,**F**) and mock infected (MI) monolayers (**C**,**D**,**G**,**H**) were immunostained with monoclonal anti-gB (GPCMV) primary antibody (Dr. Britt, UAB) + anti-mouse IgG-HRP secondary (Vectastain) (**A**,**C**,**E**,**G**) or secondary antibody only (**B**,**D**,**F**,**H**). Individual bright field images are representative of multiple fields (minimum 10 per panel). Magnification 10×. (**ii**,**iii**) Evaluation of cell-associated (CA), cell-released (CR) fractions and total virus titer at 3 days post infection (dpi) on REPI cells from 6 well plates for TAMYC (**ii**) and 22122 (**iii**).

GPCMV TAMYC strain infection of REPI cells was verified by immunohistochemical (IHC) staining of infected monolayers for gB viral protein at 4 days post infection (Figure 2i, panels A–B). TAMYC strain virus easily replicates on other non-fibroblast cell lines established in the laboratory similar to 22122 strain, including trophoblast and amniotic sac cell lines [18,19] (data not shown). TAMYC strain virus growth on REPI cells was highly cell-associated with very little cell-released virus (Figure 2ii) in contrast to 22122 strain which was 50% cell-associated and 50% cell-released virus in REPI-derived virus from infected wells of a six-well plate [19] (Figure 2iii). Importantly, TAMYC strain virus stock generated on renal epithelial cells was unable to easily replicate on fibroblast cells (Figure S2 panel A) which sharply contrasted with 22122 strain where REPI cell-derived virus stock was able to infect all available cell types including fibroblasts (Figure 2i panel E, Figure S2 panel I) [25]. Growth of TAMYC strain on fibroblast cells required extensive passage of fibroblast-infected cells over several months prior to virus being capable of replicating on fibroblasts with similar kinetics to 22122 strain. Figure S2 shows TAMYC-infected fibroblast cells at 3 days post infection using non-fibroblast-adapted (Figure S2 panel A) and TAMYC fibroblast-adapted virus stock (Figure S2 panel E) and IHC staining for gB compared to 22122 strain-infected cells (Figure S2 panel (I). A basis for this contrasting difference in virus tropism between strains awaits further evaluation but this observation is similar to HCMV clinical isolates, which require prolonged passage on fibroblasts to enable virus to adapt and this usually results in modifications of the virus compared to clinical strains. All subsequent studies for TAMYC virus were carried out on REPI cell-derived stock virus.

**Figure 3:**
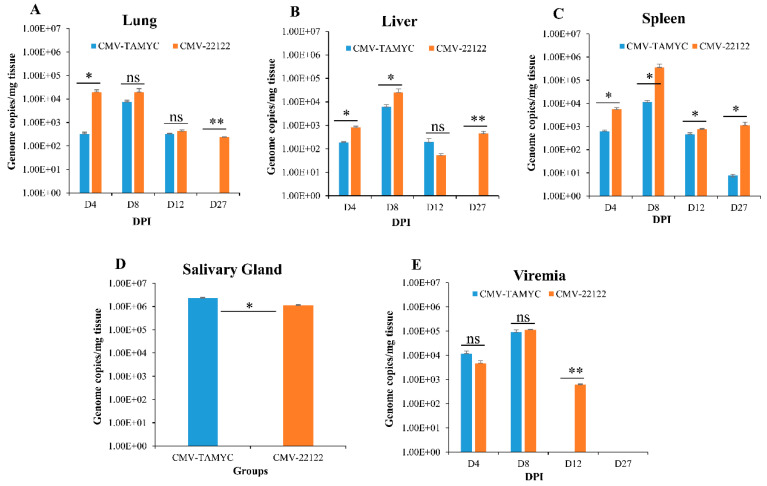
Dissemination of different GPCMV strains to target organs. Seronegative animals (*n* = 12/group) were inoculated with TAMYC (blue) or 22122 wt GPCMV (orange). At 4, 8, 12 and 27 days post infection (dpi), 3 animals from each group were evaluated for viral load in target organs: (**A**) lung; (**B**) liver; (**C**) spleen, by real-time PCR of DNA extracted from tissues. Viral load plotted as viral genome copies/mg tissue. Salivary gland tissue (**D**) was only evaluated at 27 dpi. Viremia detected at 4, 8, 12 and 27 dpi was plotted as genome copies/mL blood (**E**). Unpaired t test at 95% confidence interval * *p* < 0.05; ** below level of detection; ns = not significant.

Pathogenicity of GPCMV TAMYC in comparison to 22122 was evaluated in separate groups of seronegative animals. At day 0, animals (*n* = 12/group) were challenged with virus (10^5^ pfu/animal, SQ): TAMYC (group 1); 22122 (group 2). Subsequently, at 4, 8, 12, and 27 days post infection (dpi) animals (*n* = 3/group) were evaluated for viral load in target organs (liver, lung, spleen) and blood. At day 27 dpi, the salivary gland was additionally evaluated for viral load as well as other tissues and blood. Results are shown in Figure 3.

The TAMYC strain had roughly similar dissemination pattern to 22122 strain with ability to disseminate to all target organs (liver, lung, spleen and salivary gland) (Figure 3). TAMYC viremia was detected at early stages of infection (4–8 dpi) but 22122 strain had extended viremia (4–12 dpi), see Figure 3E. Additionally, 22122 strain was detected in all organs at 27 dpi but TAMYC was only detected in the salivary gland at 27 dpi at a similar viral load to 22122 (Figure 3). We concluded that TAMYC virus had roughly similar dissemination pattern to 22122 strain despite impaired replication on fibroblast cells demonstrating the importance of tropism to non-fibroblast cells for pathogenesis in the animal model.

### 2.2. Animal Antibody Immune Response to New Strain GPCMV (TAMYC)

**Figure 4:**
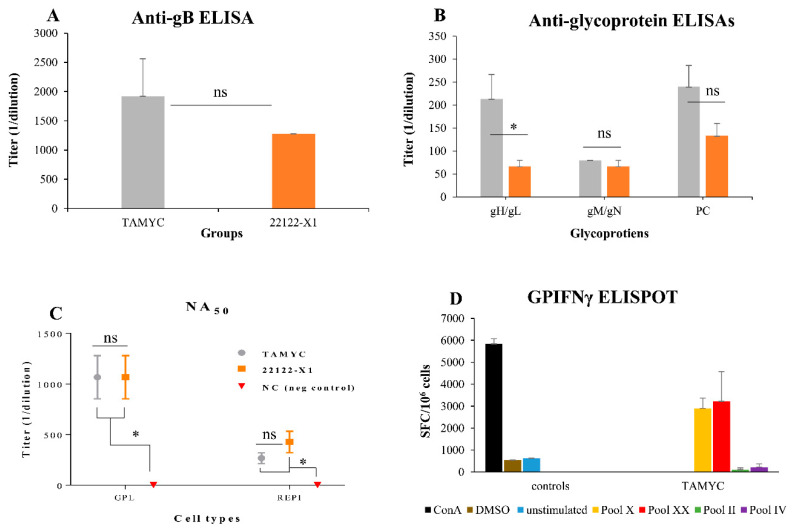
Comparative immune response of TAMYC and 22122 infected animals. Animals were infected with TAMYC (gray, *n* = 5) or 22122 GPCMV (orange, *n* = 5). Convalescent animals with comparable anti-GPCMV titer of 1:5120 were evaluated for specific antibody ELISAs to viral glycoprotein complexes. (**A**) Anti-gB ELISA titers or (**B**) antibody ELISA response to specific glycoprotein complexes (gHgL; gMgN; and PC). (**C**) Antibody neutralization (NA_50_) of wt GPCMV of TAMYC (gray circle), 22122 (orange square) or GPCMV negative (red inverted triangle) on guinea pig fibroblast lung cells (GPL) and REPI cell lines. Statistical analysis by Unpaired t test (A and B) and ANOVA (**C**) * *p* < 0.05; ns = not significant. (**D**) T cell response to GPCMV GP83 peptide pools in TAMYC strain seropositive animals determined by IFN-γ ELISPOT assay. Two reactive GP83 peptide pools, peptide pools X (yellow) and XX (red), previously identified [20], responded with splenocytes isolated from animals naturally infected with the TAMYC strain. Black, ConA positive control; brown, DMSO control; blue, unstimulated control; green, unresponsive negative (neg) peptide pool II; purple, unresponsive negative peptide pool IV. Final counts were calculated based on the number of spot-forming cells (SFC) per 10^6^ cells.

Our previous evaluations of the immune response to GPCMV has been in the context of hyperimmune animals that received multiple challenges with wild type virus, viral mutants or specific viral vaccine candidate antigens [15,20,25,29]. Commercial guinea pigs, seropositive for GPCMV TAMYC strain that were socially housed in the same holding room, had a lower immune response to all viral glycoprotein complexes compared to hyperimmune animals that received multiple injections of wild type virus (22122) over several months. In order to provide a more realistic comparison with the immune response to natural GPCMV infection, we evaluated antibody responses of guinea pigs at 12 weeks after single challenge dose (10^5^ pfu, SQ) of TAMYC, or 22122 strain. TAMYC strain seropositive animals (*n* = 8) with similar anti-GPCMV titers to 22122 infected animals (1:5120) were subsequently evaluated for antibody response by ELISA to individual glycoprotein complexes (gB, gH/gL, gM/gN and PC). Results shown in Figure 4 demonstrated that TAMYC-infected animals exhibited an antibody response to all viral glycoprotein complexes.

The highest antibody titer for a specific glycoprotein complex was to the immunodominant gB, which was slightly higher for TAMYC compared to 22122 sera but this was not statistically significant (Figure 4A). Immune responses to gM/gN and PC were similar between 22122 and TAMYC sera but the ELISA antibody titer to gH/gL was threefold higher for TAMYC sera compared to 22122 infected animal sera (1:213 v 1:67) which was statistically significant (*p* < 0.05), see Figure 4B. Overall, the antibody titers for individual glycoprotein complexes were lower for both 22122 or TAMYC strain single-dose-infected animals compared to that obtained in hyperimmune 22122-infected animals [29]. Next, pooled sera from TAMYC or 22122 strain single-dose-infected animals were evaluated on guinea pig fibroblast lung cells (GPL) and REPI epithelial cells to determine if there was a difference in ability to neutralize virus (22122 strain) infection by cell fusion PC-independent pathway (GPL) or endocytic PC-dependent pathway (REPI) [16,29]. Results demonstrated that sera from both strains were equally effective in virus neutralization on fibroblast cells (Figure 4C). However, both TAMYC and 22122 sera had weaker NA_50_ values on REPI cells (approximately three- to four-fold lower). Overall, neutralizing antibody titer from both groups was approximately fourfold lower on GPL and approximately sixfold lower on REPI cells compared to hyperimmune sera from 22122 strain animals [29]. We concluded that TAMYC and 22122 single-dose animals produced roughly similar immune responses to various target antigens but TAMYC evoked a statistically significant stronger antibody response to gH/gL and a non-statistically significant higher response to gB and PC. However, there was little difference in NA_50_ titers between TAMYC or 22122 strain animal sera. It is likely that virus neutralizing titers for guinea pig trophoblast and amniotic sac cell lines would also be similarly low compared to fibroblast NA_50_ value based on previous studies [19,25,29] but this awaits further evaluation.

In addition to antibody response to the virus, seropositive TAMYC animals were also evaluated for cell-mediated immune response to tegument protein GP83 (pp65 homolog), which has previously been demonstrated to be a T cell target antigen in GPCMV studies [20,35]. An IFN-γ ELISPOT of splenocytes of GPCMV TAMYC convalescent animals demonstrated a response to specific GP83 peptides (Figure 4D) and confirmed that convalescent animals had a response to GP83 as previously noted for animals infected with GPCMV 22122 strain [20,29]. We concluded that convalescent TAMYC strain-infected animals had a roughly similar immune response to GP83 as previously noted for 22122 strain.

### 2.3. Natural Immunity and Cross Strain Protection against Reinfection

**Figure 5:**
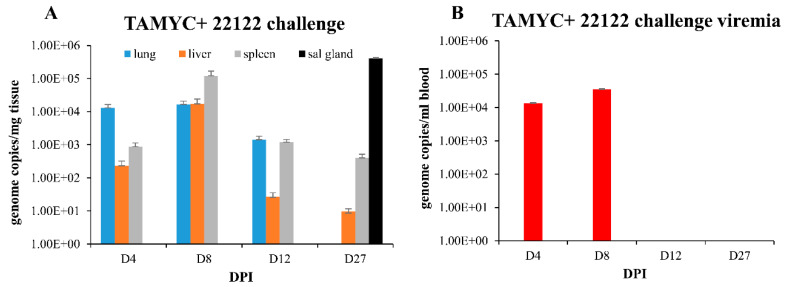
GPCMV pathogenesis in seropositive animals (TAMYC) challenged with heterologous virus strain (22122). Convalescent TAMYC seropositive animals (*n* = 12) were challenged with 22122 GPCMV, 10^5^ pfu SQ. At 4, 8, 12, and 27 dpi, 3 animals per group were evaluated for viral load in target organs by real-time PCR and plotted as viral genome copies/mg tissue (**A**). Salivary gland tissue was only evaluated at day 27. Viremia evaluated at 4, 8, 12, and 27 dpi plotted as genome copies/mL blood (**B**).

A recent 22122 strain GPCMV DISC vaccine was highly effective in protection against challenge by wild type 22122 strain and prevented challenge virus dissemination [29], unlike a full length gB vaccine strategy [25]. A prerequisite of a successful vaccine strategy against congenital CMV is the ability to provide protection against multiple strains of CMV. However, all GPCMV vaccine studies to date have only evaluated vaccine efficacy against prototype 22122 strain in homogenous strain studies. Consequently, it was important to determine the level of cross protection provided by convalescent immunity to establish a minimum level of expectation for a successful vaccine strategy against multiple GPCMV strains. A group of animals (*n* = 12) convalescent for TAMYC strain infection and matched for anti-GPCMV titer (1:5120) were challenged with 22122 strain GPCMV (10^5^ pfu, SQ). At various time points (4, 8, 12 and 27 dpi), animals (*n* = 3/group) were euthanized and viral load in target organs (liver, lung, spleen and blood) was evaluated. At 27 dpi the viral load in the salivary gland tissue was additionally evaluated. Results in Figure 5A demonstrated that virus dissemination to target organs was observed in lung, liver and spleen on days 4–12 and in liver and spleen on day 27. Additionally, convalescent immunity did not prevent virus infection of the salivary gland at day 27 dpi (Figure 5A).

Convalescent immunity did shorten viremia, which was detected on days 4–8 dpi at comparable levels but absent at 12 dpi (Figure 5B) compared to seronegative animals infected with 22122 strain [16]. Therefore, pre-existing immunity to GPCMV (TAMYC strain) had limited ability to cross protect against heterogenous 22122 strain GPCMV challenge. We concluded that although convalescent immunity of GPCMV seropositive animals can provide sterilizing immunity to the homogenous challenge of a viral strain [29], this requires a robust immune response observed in hyperimmune animals [29]. Immunity to re-infection in heterogenous virus challenge (TAMYC strain seropositive and 22122 strain challenge) is more limited as heterogenous viruses remained capable of disseminating to target organs and the salivary glands. This potentially indicates a limitation of adaptive immune response evoked in virus-infected convalescent animals. Possibly, the same limitation might also apply to the GPCMV DISC vaccine strategy [29], but cross strain protection remains to be evaluated.

### 2.4. Natural Immunity and Cross Strain Protection against Congenital Infection

Currently, in the guinea pig model, only a PC+ DISC vaccine strategy has been demonstrated to provide complete protection against cCMV [29]. However, the DISC vaccine as well as all other vaccine studies performed by various investigators in the guinea pig model have only evaluated protection against cCMV by GPCMV strain 22122 and no other strain. Since multiple strains of HCMV enable the potential for re-infection despite convalescent immunity, vaccine-based cross strain protection should be evaluated as part of vaccine efficacy studies and especially at the preclinical stage. In order to establish a base line for cross protective immunity against cCMV between strains, we evaluated the ability of convalescent TAMYC strain-seropositive female animals to provide cross protection against a heterologous challenge virus strain (22122) during pregnancy. Convalescent TAMYC strain-seropositive female animals (*n* = 8, group 1) matched for anti-GPCMV titer (1:5120) were mated with seronegative males and at day 30–35 of gestation (mid–late second trimester) challenged with wild type salivary gland stock 22122 strain GPCMV (10^5^ pfu, SQ). A control group of seronegative female animals (*n* = 15, group 2) were mated and similarly challenged with 22122 strain GPCMV (10^5^ pfu, SQ) at day 30–35 of gestation. All animals were allowed to proceed to term. Pups were evaluated for viral load in target organs (liver, lung, spleen and brain). The overall outcome of the study is shown in Table 2. All group 1 pups (seropositive, convalescent TAMYC) were born live. In contrast, the control group 2 (seronegative) animals, had both live and still-born animals present in litters with a total of 56.2% live pups and 43.8% still-born dead pups (Table 2).

Evaluation of viral load in pups from both groups (Table 3) identified 4/33 pups in group 1 with detectable viral load in one or more target organs, in contrast to 28/35 pups in the control group 2 with detectable viral load in one or more target organs (Table 3). Overall, convalescent immunity reduced the cCMV transmission rate from 80% in the control to 12% in group 1, seropositive TAMYC convalescent dams (Table 3). Additionally, pups from group 1 with detectable viral load had lower levels in the target organs compared to control group 2 pups (Table 3). Overall, despite an inability of convalescent immunity to prevent challenge virus dissemination in dams (Figure 5), immune response was sufficient to substantially reduce the risk of cCMV. We concluded that an ideal minimum expectation of a successful vaccine strategy in this model would be to provide high-level protection against cCMV by a heterogenous virus challenge in addition to high efficacy against a homogenous strain.

## 3. Discussion

Candidate CMV vaccines should be evaluated for efficacy in a relevant preclinical animal model but studies are complicated by the requirement of a species-specific animal CMV. A further limitation is that there are only two models for cCMV (rhesus macaque and guinea pig). Although no cCMV vaccine protection studies have been evaluated in the NHP model, it is potentially more relevant to HCMV disease. Various vaccines have been evaluated against horizontal virus transmission for RhCMV. Both gB and PC vaccines have separately failed to prevent RhCMV natural infection, despite robust immune response, which potentially indicates a limitation of approaches focused on an individual glycoprotein complex [43,44]. RhCMV T cell target antigens (IE1 and pp65 homologs) have also failed to prevent natural infection [45]. A complication of NHP studies is the limited number of RhCMV seronegative rhesus macaques. Consequently, the guinea pig is perhaps the most realistic animal model to evaluate cCMV vaccine strategies in a high throughput approach. However, the model is not without specific shortcomings including reagents and assays. We have developed a number of novel cell lines and assays to enable evaluation of virus tropism and host immune responses [15,16,18,19,20,25,29,30].

A potentially important limitation of the current guinea pig model is the use of a single viral strain of GPCMV, which was passaged on fibroblast cells multiple times prior to ATCC deposition [37]. Consequently, despite the ability of 22122 strain from salivary gland virus stock to cause cCMV, an underlying concern is that this strain might not truly resemble a wild type virus and additionally if immune response was cross protective between strains. Therefore, studies with an additional GPCMV strain would seem logical, especially since numerous HCMV strains exist [46]. The novel strain of GPCMV (TAMYC) fulfills the criteria of a new strain by exhibiting altered predicted sequence for gO protein (GP74) compared to 22122 strain as well as other glycoprotein sequences. Variability in sequence is commonly observed in HCMV strains, especially in gO with up to 30% difference between strains [38,39,40,41,47]. In the case of GPCMV, TAMYC and 22122, strains exhibited 25% difference in the gO amino acid sequence and importantly two N-terminal region glycosylation sites were missing, which potentially alters ability of the viral trimer (gH/gL/gO) to interact with cell receptor PDGFRA which can be influenced by glycosylation [30]. In HCMV, gO polymorphisms modify virus cell-free and cell-to-cell spread [48] and this might account for the basis of the TAMYC strain being highly cell-associated but this requires further study. Potentially, there are additional differences between the two GPCMV strains but this also awaits further evaluation.

Phenotypically, it is important to note that unlike 22122, the TAMYC strain was extremely cell-associated and grew poorly on fibroblasts compared to growth on epithelial cells unless TAMYC was extensively adapted to fibroblasts. In contrast, 22122 strain virus stock generated on REPI cells grows equally well on fibroblasts and various non-fibroblast cell lines [18,19,29]. In this regard, the new GPCMV strain more closely resembles a low pass HCMV clinical isolate [49]. Altered tropism might be a result of modified ratio of PC to gH/gL/gO trimer between strains and/or perhaps differential expression of a homolog UL148 [50] but these aspects await future study. An additional novel GPCMV strain (designated CIDMTR) was recently identified [51] and TAMYC differs from this strain with 87% identity for gO protein based on ClustalW protein alignment of predicted amino acid sequence [51] (unpublished observation McGregor and Choi). Therefore, based on HCMV gO strain genotypes, which vary between 10 and 30%, TAMYC should be considered a separate strain to CIDMTR as well as 22122. A specific concern related to this additional reported CIDMTR strain is that it was likely passed on fibroblast cells and so potentially incurred modifications during adaptation and so may have limited advantage in use over the existing 22122 strain. In HCMV, initial mutation and virus adaptation can occur within one pass on fibroblast cells but other modifications can occur with additional passes [52]. Another GPCMV strain isolate was also reported by Cardin and colleagues [53]. Unfortunately, immune response or virus tropism associated with any other new strain of GPCMV awaits specific study so further comparisons are not possible. It is interesting to note that in MCMV, despite differences in DNA sequence between strains, there is limited or no variation in glycoprotein amino acid sequence between strains. In the case of Smith and K181 strains of MCMV, M74 (gO) exhibit 100% homology at the protein level [54]. Therefore, GPCMV would appear to differ from MCMV as novel strains carry protein polymorphisms.

In HCMV, because of the risk of re-infection by a new strain of virus, a vaccine needs to provide protection that exceeds convalescent natural immunity. In homogenous virus challenge studies of convalescent animals, it appears that in the guinea pig model, protection against re-infection by the same strain of virus is possible but the immune response must be greater than that of natural immunity as only hyperimmune animals or DISC(PC+) multi-vaccinated animals are protected from re-infection [29]. Antibodies to HCMV gB can constitute 40–70% neutralizing activity to HCMV in convalescent patients [55,56] but a gB vaccine strategy provided approximately 50% efficacy in clinical trials [57,58]. GPCMV gB is essential for infection of both fibroblast and non-fibroblast cell types [15,16,19] but gB vaccine strategies against 22122 strain provided about 50% efficacy against cCMV. Previous GPCMV gB vaccine studies had utilized truncated gB protein. Our recent studies with a full length gB Ad vector strategy [25] demonstrated that full length gB with an ability to form a higher-order trimer complex generated a higher neutralizing antibody titer on fibroblast cells. However, full length gB vaccine sera were less effective for neutralization on non-fibroblast cells compared to convalescent sera with antibodies to the PC. This would suggest that a gB vaccine strategy would have limited ability to cross protect against multiple strains, especially if the virus preferentially infected non-fibroblast cells as is the case for TAMYC strain. However, the Ad vector gB vaccine did not prevent homogenous virus dissemination. Consequently, results would suggest that a gB vaccine would be limited in protecting against infection by additional strains despite relatively high identity between strains. It is possible that a PC vaccine strategy might also be limited in protection against GPCMV infection and we are currently evaluating PC specific vaccine strategies. In HCMV, the antibody response to the PC has been shown to be important for broad spectrum neutralization of clinical strains on various cell types [32].

In conclusion, it should be an expectation of any strategy against cCMV that it meets or exceeds protection levels attained by convalescent immunity because of the risk of re-infection by new strains of CMV. In this current study, we have isolated a novel strain of GPCMV and demonstrated that although convalescent immunity does not protect against re-infection by a heterogenous strain challenge, the existing immunity is sufficient to provide relatively high level of protection against cCMV by heterogenous challenge virus. Consequently, this outcome indicates a likely important standard that any vaccine against cCMV should meet and ideally exceed. Undoubtedly, the new strain of GPCMV will be important in future vaccine research. Overall, these studies demonstrate that GPCMV has potential strain diversity similar to HCMV and further emphasizes the importance of this model for translational studies.

## 4. Materials and Methods

### 4.1. Virus, Cells, Synthetic Genes and Oligonucleotides

Wild type GPCMV (strain 22122, ATCC VR682 or new strain isolate, designated TAMYC) propagated on guinea pig fibroblast lung cells (GPL; ATCC CCL 158) and REPI cell line as previously described [16,18]. Virus stocks for antibody neutralization assays were generated on REPI cells. Virus titers were determined by GPCMV titration on REPI and fibroblast cells [59]. Recombinant defective adenovirus (Ad5) vectors encoding GPCMV glycoproteins (gB, gH, gL, GP129, GP131 and GP133) were previously described [15,16,25]. Oligonucleotides were synthesized by Sigma-Genosys (The Woodlands, TX, USA).

### 4.2. Ethics and Animal Studies

Guinea pig (Hartley) animal studies were performed under guidance and permission by Texas A&M University animal use review committee, IACUC (Texas A&M University, College Station, TX, USA), under permit 2017-0227 granted in 2017 to AM. All study procedures were carried out in strict accordance with the recommendations in the “Guide for the Care and Use of Laboratory Animals of the National Institutes of Health.” Animals were observed daily by trained animal care staff, and animals that required care were referred to the attending veterinarian for immediate care or euthanasia. Terminal euthanasia was carried out by lethal CO_2_ overdose followed by cervical dislocation in accordance with IACUC protocol and NIH guidelines. Animals purchased from Charles River Laboratories were screened for GPCMV by anti-GPCMV ELISA of sera collected by toenail clip bleed as previously described [15]. Animal studies were performed to evaluate (a) immune response to GPCMV infection, (b) virus dissemination in seropositive and seronegative animals, (c) congenital GPCMV in seropositive and seronegative animals. Experiment 1. GPCMV strain dissemination (TAMYC) was carried out on seronegative animals. On day 0, animals were challenged with virus (10^5^ pfu, SQ). At various days post infection, 3 animals were euthanized and viral load determined for target organs and blood at 4, 8, 12, and 27 dpi as previously described [29]. Comparative study was carried out on an additional group of animals for 22122 virus strain as described above. Experiment 2. Convalescent immunity (TAMYC) and cross strain protection by heterogenous challenge (22122) were assessed. Twelve animals convalescent for TAMYC matched for anti-GPCMV titer were challenged with 22122 strain virus (10^5^ pfu, SQ) and viral load evaluated for target organs and blood at 4, 8 12 and 27 dpi. Seronegative animals challenged with 22122 strain in experiment 1 served as the control group. Experiment 3. Congenital GPCMV in seropositive and seronegative animals was analyzed. Animals convalescent for TAMYC strain (*n* = 8, Group 1) matched for anti-GPCMV titer (1:5120) were mated with seronegative males and at day 30–35 of gestation (mid-late 2nd trimester) challenged with wild type salivary gland stock 22122 strain GPCMV (10^5^ pfu, SQ). A control group of seronegative female animals (*n* = 15, Group 2) were mated and similarly challenged with 22122 strain GPCMV at day 30–35 of gestation. Animals proceeded to term and pups were evaluated for viral load in target organs (liver, lung, spleen and brain).

### 4.3. Isolation of a New Strain of GPCMV

Guinea pigs purchased under permit 2017-0227 granted in 2017 to AM by IACUC (Texas A&M University) were sourced from the same holding room from a commercial vendor and screened by toe nail clip bleed for GPCMV sera status by anti-GPCMV ELISA [15]. Animals seropositive for GPCMV were euthanized and salivary gland tissue and spleen isolated, homogenized and a portion used to evaluate viral load by real time PCR for individual animals. Positive tissue homogenates (salivary gland) from individual animals were separately co-cultured on REPI cells. Virus studies are based on new strain GPCMV isolated from one animal (TAMYC) and virus passaged once on REPI cells prior to storage at −80 °C as P1 REPI stock. TAMYC is an anagram of the institute where the virus was isolated (Texas A&M) and the investigators that isolated the virus (AM and YC). TAMYC viral DNA isolated from P1-infected REPI cells was used as template for PCR cloning of TAMYC glycoprotein genes into TA cloning vectors as previously described [16]. Individual GPCMV glycoprotein genes (Table 1) were PCR amplified with primers shown in Table S1 (see supplementary data) or using primers previously published [15]. DNA sequence of 3 independent bacterial clones for each gene was determined and subsequently assembled and analyzed in comparison to 22122 strain GPCMV genes by MacVector software. Predicted glycoprotein amino acid sequences for 22122 and TAMYC were compared by ClustalW protein alignment by MacVector software. Additional analysis was made to the CIDMTR GPCMV strain [51] (data not shown). DNA sequencing was carried out by Functional Biosciences and Eurofins Genomics.

### 4.4. GPCMV Glycoprotein ELISAs

Specific glycoprotein complex ELISAs were carried out as previously described using positive coating antigen derived from renal epithelial cell monolayers transduced with recombinant replication-defective adenovirus (Ad) vectors expressing specific glycoprotein complexes or control recombinant Ad vector expressing GFP for negative coating antigen [15,16,20,25]. Except for the case of gM/gN ELISA which utilized transient expression, plasmids with synthetic codons optimized expression plasmids for transfection onto guinea pig cells [15]. Harvested cells were washed with PBS and cell pellets fixed prior to processing as coating antigen. Protein concentration was normalized by Bradford assay. MaxiSorp ELISA plates (NUNC) were coated with 1.0 µg of either Ag+ or Ag^−^ preparations diluted in carbonate coating buffer overnight at 4 °C, washed in 1X PBST then blocked with 2% nonfat dry milk. Test sera were diluted in blocking buffer from 1:40 to 1:20480 in doubling dilutions, incubated for 2 h at 37 °C and then reacted with anti-Guinea Pig IgG peroxidase antibody (Sigma) diluted (1:1000) in blocking buffer for an additional 1 h at 37 °C before reacting with TMB membrane peroxidase substrate (KPL). Net optical density (OD) (absorbance 450 nm) was attained by subtracting OD of Ag^−^ from OD of Ag+. All ELISAs described in this report were carried out with the same batch of coating antigen. The described approach is based on similar strategies for glycoprotein complex expression for HCMV and RhCMV and ELISAs [60,61]. All ELISAs were run a minimum of three times in duplicates. ELISA reactivity was considered positive if the net OD was greater than, or equal to 0.2, as determined by GPCMV-negative serum. Final antibody titer was determined as the reciprocal of the highest serum dilution above the background OD 0.2 ABS.

### 4.5. GPCMV Neutralization Assays

GPCMV neutralization assays (NA_50_) were performed on GPL fibroblasts and REPI cells with PC+ GPCMV (22122 strain) virus [15,16] using pooled sera from a specific group of animals (22122 seropositive, TAMYC seropositive or control seronegative) as previously described [20]. Serially diluted sera were incubated with GPCMV in media containing 1% rabbit complement (Equitech Bio, Kerr County, TX, USA) for 90 min at 37 °C before infecting cells for 1 h. Infected cells and supernatant were collected on day 4 then titrated on GPLs. Final neutralizing antibody titer was the inverse of the highest dilution producing 50% or greater reduction in plaques compared to virus only control. NA_50_ assays were performed from each sample three times concurrently with the same virus stocks between groups.

### 4.6. Guinea Pig Interferon Gamma Enzyme-Linked Immunospot (ELISPOT) Assay

The guinea pig IFN-γ ELISPOT assays were performed following a previously described protocol using freshly isolated splenocytes and GPCMV GP83 peptide pools [20]. Anti-guinea pig IFN-γ monoclonal antibodies (V-E4 and N-G3) were previously characterized [62]. Briefly, PVDF membrane 96-well plates were coated with guinea pig IFN-γ capture antibody, V-E4, and incubated overnight at 4 °C, blocked then freshly isolated splenocytes added before being exposed to GPCMV GP83 9 mer peptide pools and incubated for 18 h. Biotinylated detection antibody, N-G3 was added before streptavidin-AP conjugate (R&D Systems) then detected with BCIP/NBT (Life Technologies, Carlsbad, CA, USA). Membranes were dried before spots were counted on ImmunoSpot S6 (CTL). Final counts were calculated based on spot-forming cells (SFC) per 10^6^ cells after background spots (cells only without any stimulation) were subtracted. ConA (10 µg/mL) was used as positive control and other controls included cells only control, DMSO control (peptide background), and nonspecific peptide control and media only control.

### 4.7. Real Time PCR

Blood and tissues (lung, liver, spleen, salivary gland) were collected from euthanized guinea pigs to determine the viral load as previously described [15,20]. Briefly, for tissue DNA extraction, FastPrep 24 (MP Biomedical, Santa Ana, CA, USA) was used to homogenize tissues as a 20% weight/volume homogenate in Lysing Matrix D (MP Biomedicals). To obtain DNA from whole blood, blood was collected into tubes containing ACD anticoagulant and 200 μL of blood was subsequently used per extraction. DNA was extracted using the QIAcube HT (Qiagen, Hilden, Germany) according to manufacturer’s blood or tissue protocols. Viral load was determined by real time PCR on LightCycler 480 (Roche Applied Science, Penzberg, Germany) using primers and hydrolysis probe to amplify a product from the GPCMV GP44 gene. Data was collected by “single” acquisition during the extension step. Standard curve was generated using GPCMV GP44 plasmid [63] for quantification and assay sensitivity. The sensitivity of the assay was determined to be 5 copies/reaction. Viral load was expressed as genome copy number/mL of blood or genome copy number/mg tissue. Results calculated were a mean value of triplicate PCR runs per sample.

### 4.8. Statistical Analysis 

All statistical analyses were conducted with GraphPad Prism (version 7) software. Replicate means were analyzed using one-way analysis of variance (ANOVA), Unpaired t test at 95% Confidence interval, or Fisher’s exact test, with significance taken as a *p* value of <0.05 or as specified in the figure legends.

## Figures and Tables

**Table 1:** Nucleic acid and amino acid homology comparison between guinea pig cytomegalovirus (GPCMV) TAMYC and 22122 strains.

Glycoproteins	Nucleic Acid (%) Homology	Amino Acid (%) Homology
GP55 (gB)	99	99
GP73 (gN)	95	92
GP74 (gO)	84	75
GP75 (gH)	85	84
GP100 (gM)	98	99
GP115 (gL)	99	98
GP129	90	88
GP131	86	89
GP133	91	91

**Table 2:** Congenital infection outcomes for live versus dead pups.

	Group 1 Seropositive	Group 2 Seronegative
Pregnant	8/8 (100%)	15/15 (100%)
Litters delivered	8	14 **^a^**
Litters with only live pups	8	7
Litters with mix (live and dead) pups	0	3
Litters with only dead pups	0	4
Total pups (live) * Pups evaluated by PCR	33 (100%) 33	27 (56.25%) 24 **^b^**
Total pups (dead) * Pups evaluated by PCR	0 (0.00%) 0	21 (43.75%) 11 **^c^**

* *p* = 0.0001 Fisher’s exact test; **^a^** 1 dam died during pregnancy on day 8 post challenge; **^b^** 3 live pups were not evaluated by PCR due to premature birth (sub day 12 post challenge); **^c^** 10 dead pups were not evaluated by PCR due to premature birth (sub day 12 post challenge).

**Table 3:** Congenital infection outcome and mean viral load (genome copies/mg tissue) based on cytomegalovirus (CMV) detection in target tissues of pups.

Vaccine	Lung	Liver	Spleen	Brain	CMV + Pups
Group 1 Seropositive	3/33 * (9.1%) 2.43 × 10^2^	1/33 * (3.0%) 3.6 × 10^2^	1/33 * (3.0%) 1.52 × 10^2^	1/33 * (3.0%) 1.38 × 10^2^	4/33 * (12.12%)
Group 2 Seronegative	21/35 (60.0%) 4.62 × 10^2^	15/35 (42.9%) 4.25 × 10^2^	19/35 (54.3%) 1.33 × 10^3^	18/35 (51.4%) 1.87 × 10^3^	28/35 (80.00%)

* *p* = 0.0001 Statistical analysis comparing number of CMV-positive organs in each group determined by Fisher’s exact test.

## Supplementary Materials

Supplementary materials can be found at https://www.mdpi.com/1422-0067/21/17/5997/s1.

## References

[1] Ross S.A., Boppana S.B. (2005). Congenital cytomegalovirus infection: Outcome and diagnosis. Semin. Pediatr. Infect. Dis..

[2] Griffiths P.D., Walter S. (2005). Cytomegalovirus. Curr. Opin. Infect. Dis..

[3] Fowler K.B., Dahle A.J., Boppana S.B., Pass R.F. (1999). Newborn hearing screening: Will children with hearing loss caused by congenital cytomegalovirus infection be missed?. J. Pediatr..

[4] Manicklal S., Emery V.C., Lazzarotto T., Boppana S.B., Gupta R.K. (2013). The “silent” global burden of congenital cytomegalovirus. Clin. Microbiol. Rev..

[5] Fowler K.B., Stagno S., Pass R.F. (2003). Maternal immunity and prevention of congenital cytomegalovirus infection. JAMA.

[6] Schleiss M.R., Permar S.R., Plotkin S.A. (2017). Progress toward Development of a Vaccine against Congenital Cytomegalovirus Infection. Clin. Vaccine Immunol..

[7] Pass R.F., Zhang C., Evans A., Simpson T., Andrews W., Huang M.L., Corey L., Hill J., Davis E., Flanigan C. (2009). Vaccine prevention of maternal cytomegalovirus infection. N. Engl. J. Med..

[8] Tabata T., Petitt M., Fang-Hoover J., Freed D.C., Li F., An Z., Wang D., Fu T.M., Pereira L. (2019). Neutralizing Monoclonal Antibodies Reduce Human Cytomegalovirus Infection and Spread in Developing Placentas. Vaccines.

[9] Sylwester A.W., Mitchell B.L., Edgar J.B., Taormina C., Pelte C., Ruchti F., Sleath P.R., Grabstein K.H., Hosken N.A., Kern F. (2005). Broadly targeted human cytomegalovirus-specific CD4+ and CD8+ T cells dominate the memory compartments of exposed subjects. J. Exp. Med..

[10] Yue Y., Barry P.A. (2008). Rhesus cytomegalovirus a nonhuman primate model for the study of human cytomegalovirus. Adv. Virus Res..

[11] Griffith B.P., McCormick S.R., Fong C.K., Lavallee J.T., Lucia H.L., Goff E. (1985). The placenta as a site of cytomegalovirus infection in guinea pigs. J. Virol..

[12] Kaufmann P., Benirschke K. (2004). Guinea Pig Cavia procellus. Comparitive Placentation.

[13] Mess A. (2007). The Guinea pig placenta: Model of placental growth dynamics. Placenta.

[14] Woolf N.K., Koehrn F.J., Harris J.P., Richman D.D. (1989). Congenital cytomegalovirus labyrinthitis and sensorineural hearing loss in guinea pigs. J. Infect. Dis..

[15] Coleman S., Hornig J., Maddux S., Choi K.Y., McGregor A. (2015). Viral Glycoprotein Complex Formation, Essential Function and Immunogenicity in the Guinea Pig Model for Cytomegalovirus. PLoS ONE.

[16] Coleman S., Choi K.Y., Root M., McGregor A. (2016). A Homolog Pentameric Complex Dictates Viral Epithelial Tropism, Pathogenicity and Congenital Infection Rate in Guinea Pig Cytomegalovirus. PLoS Pathog..

[17] Auerbach M., Yan D., Fouts A., Xu M., Estevez A., Austin C.D., Bazan F., Feierbach B. (2013). Characterization of the guinea pig CMV gH/gL/GP129/GP131/GP133 complex in infection and spread. Virology.

[18] Coleman S., Choi K.Y., McGregor A. (2017). Cytomegalovirus UL128 homolog mutants that form a pentameric complex produce virus with impaired epithelial and trophoblast cell tropism and altered pathogenicity in the guinea pig. Virology.

[19] Choi K.Y., El-Hamdi N.S., McGregor A. (2020). Requirements for guinea pig cytomegalovirus tropism and antibody neutralization on placental amniotic sac cells. J. Gen. Virol..

[20] Choi K.Y., Root M., McGregor A. (2016). A Novel Non-Replication-Competent Cytomegalovirus Capsid Mutant Vaccine Strategy Is Effective in Reducing Congenital Infection. J. Virol..

[21] Britt W.J., Harrison C. (1994). Identification of an abundant disulfide-linked complex of glycoproteins in the envelope of guinea pig cytomegalovirus. Virology.

[22] Schleiss M.R., Jensen N.J. (2003). Cloning and expression of the guinea pig cytomegalovirus glycoprotein B (gB) in a recombinant baculovirus: Utility for vaccine studies for the prevention of experimental infection. J. Virol. Methods.

[23] Schleiss M.R., Bourne N., Stroup G., Bravo F.J., Jensen N.J., Bernstein D.I. (2004). Protection against congenital cytomegalovirus infection and disease in guinea pigs, conferred by a purified recombinant glycoprotein B vaccine. J. Infect. Dis..

[24] Hashimoto K., Yamada S., Katano H., Fukuchi S., Sato Y., Kato M., Yamaguchi T., Moriishi K., Inoue N. (2013). Effects of immunization of pregnant guinea pigs with guinea pig cytomegalovirus glycoprotein B on viral spread in the placenta. Vaccine.

[25] Choi K.Y., El-Hamdi N.S., McGregor A. (2020). Neutralizing antibodies to gB based CMV vaccine requires full length antigen but reduced virus neutralization on non-fibroblast cells limits vaccine efficacy in the guinea pig model. Vaccine.

[26] Cardin R.D., Bravo F.J., Pullum D.A., Orlinger K., Watson E.M., Aspoeck A., Fuhrmann G., Guirakhoo F., Monath T., Bernstein D.I. (2016). Replication-defective lymphocytic choriomeningitis virus vectors expressing guinea pig cytomegalovirus gB and pp65 homologs are protective against congenital guinea pig cytomegalovirus infection. Vaccine.

[27] Swanson E.C., Gillis P., Hernandez-Alvarado N., Fernandez-Alarcon C., Schmit M., Zabeli J.C., Wussow F., Diamond D.J., Schleiss M.R. (2015). Comparison of monovalent glycoprotein B with bivalent gB/pp65 (GP83) vaccine for congenital cytomegalovirus infection in a guinea pig model: Inclusion of GP83 reduces gB antibody response but both vaccine approaches provide equivalent protection against pup mortality. Vaccine.

[28] Wu Y., Prager A., Boos S., Resch M., Brizic I., Mach M., Wildner S., Scrivano L., Adler B. (2017). Human cytomegalovirus glycoprotein complex gH/gL/gO uses PDGFR-alpha as a key for entry. PLoS Pathog..

[29] Choi K.Y., El-Hamdi N.S., McGregor A. (2019). Inclusion of the Viral Pentamer Complex in a Vaccine Design Greatly Improves Protection against Congenital Cytomegalovirus in the Guinea Pig Model. J. Virol..

[30] El-Hamdi N.S., Choi K.Y., McGregor A. (2020). Guinea pig cytomegalovirus trimer complex gH/gL/gO uses PDGFRA as universal receptor for cell fusion and entry. Virology.

[31] Freed D.C., Tang Q., Tang A., Li F., He X., Huang Z., Meng W., Xia L., Finnefrock A.C., Durr E. (2013). Pentameric complex of viral glycoprotein H is the primary target for potent neutralization by a human cytomegalovirus vaccine. Proc. Natl. Acad. Sci. USA.

[32] Ha S., Li F., Troutman M.C., Freed D.C., Tang A., Loughney J.W., Wang D., Wang I.M., Vlasak J., Nickle D.C. (2017). Neutralization of Diverse Human Cytomegalovirus Strains Conferred by Antibodies Targeting Viral gH/gL/pUL128-131 Pentameric Complex. J. Virol..

[33] Chiuppesi F., Wussow F., Johnson E., Bian C., Zhuo M., Rajakumar A., Barry P.A., Britt W.J., Chakraborty R., Diamond D.J. (2015). Vaccine-Derived Neutralizing Antibodies to the Human Cytomegalovirus gH/gL Pentamer Potently Block Primary Cytotrophoblast Infection. J. Virol..

[34] McGregor A., Liu F., Schleiss M.R. (2004). Molecular, biological, and in vivo characterization of the guinea pig cytomegalovirus (CMV) homologs of the human CMV matrix proteins pp71 (UL82) and pp65 (UL83). J. Virol..

[35] Schleiss M.R., Lacayo J.C., Belkaid Y., McGregor A., Stroup G., Rayner J., Alterson K., Chulay J.D., Smith J.F. (2007). Preconceptual administration of an alphavirus replicon UL83 (pp65 homolog) vaccine induces humoral and cellular immunity and improves pregnancy outcome in the guinea pig model of congenital cytomegalovirus infection. J. Infect. Dis..

[36] Wang D., Freed D.C., He X., Li F., Tang A., Cox K.S., Dubey S.A., Cole S., Medi M.B., Liu Y. (2016). A replication-defective human cytomegalovirus vaccine for prevention of congenital infection. Sci. Transl. Med..

[37] Hartley J.W., Rowe W.P., Huebner R.J. (1957). Serial propagation of the guinea pig salivary gland virus in tissue culture. Proc. Soc. Exp. Biol. Med..

[38] Zhou M., Yu Q., Wechsler A., Ryckman B.J. (2013). Comparative analysis of gO isoforms reveals that strains of human cytomegalovirus differ in the ratio of gH/gL/gO and gH/gL/UL128-131 in the virion envelope. J. Virol..

[39] Rasmussen L., Geissler A., Cowan C., Chase A., Winters M. (2002). The genes encoding the gCIII complex of human cytomegalovirus exist in highly diverse combinations in clinical isolates. J. Virol..

[40] Stanton R., Westmoreland D., Fox J.D., Davison A.J., Wilkinson G.W. (2005). Stability of human cytomegalovirus genotypes in persistently infected renal transplant recipients. J. Med. Virol..

[41] Mattick C., Dewin D., Polley S., Sevilla-Reyes E., Pignatelli S., Rawlinson W., Wilkinson G., Dal Monte P., Gompels U.A. (2004). Linkage of human cytomegalovirus glycoprotein gO variant groups identified from worldwide clinical isolates with gN genotypes, implications for disease associations and evidence for N-terminal sites of positive selection. Virology.

[42] Stegmann C., Rothemund F., Laib Sampaio K., Adler B., Sinzger C. (2019). The N Terminus of Human Cytomegalovirus Glycoprotein O Is Important for Binding to the Cellular Receptor PDGFRalpha. J. Virol..

[43] Wussow F., Yue Y., Martinez J., Deere J.D., Longmate J., Herrmann A., Barry P.A., Diamond D.J. (2013). A vaccine based on the rhesus cytomegalovirus UL128 complex induces broadly neutralizing antibodies in rhesus macaques. J. Virol..

[44] Abel K., Martinez J., Yue Y., Lacey S.F., Wang Z., Strelow L., Dasgupta A., Li Z., Schmidt K.A., Oxford K.L. (2011). Vaccine-induced control of viral shedding following rhesus cytomegalovirus challenge in rhesus macaques. J. Virol..

[45] Yue Y., Wang Z., Abel K., Li J., Strelow L., Mandarino A., Eberhardt M.K., Schmidt K.A., Diamond D.J., Barry P.A. (2008). Evaluation of recombinant modified vaccinia Ankara virus-based rhesus cytomegalovirus vaccines in rhesus macaques. Med. Microbiol. Immunol..

[46] Suarez N.M., Wilkie G.S., Hage E., Camiolo S., Holton M., Hughes J., Maabar M., Vattipally S.B., Dhingra A., Gompels U.A. (2019). Human Cytomegalovirus Genomes Sequenced Directly from Clinical Material: Variation, Multiple-Strain Infection, Recombination, and Gene Loss. J. Infect. Dis..

[47] Renzette N., Bhattacharjee B., Jensen J.D., Gibson L., Kowalik T.F. (2011). Extensive genome-wide variability of human cytomegalovirus in congenitally infected infants. PLoS Pathog..

[48] Day L.Z., Stegmann C., Schultz E.P., Lanchy J.M., Yu Q., Ryckman B.J. (2020). Polymorphisms in Human Cytomegalovirus Glycoprotein O (gO) Exert Epistatic Influences on Cell-Free and Cell-to-Cell Spread and Antibody Neutralization on gH Epitopes. J. Virol..

[49] Murrell I., Bedford C., Ladell K., Miners K.L., Price D.A., Tomasec P., Wilkinson G.W.G., Stanton R.J. (2017). The pentameric complex drives immunologically covert cell-cell transmission of wild-type human cytomegalovirus. Proc. Natl. Acad. Sci. USA.

[50] Nguyen C.C., Siddiquey M.N.A., Zhang H., Li G., Kamil J.P. (2018). Human Cytomegalovirus Tropism Modulator UL148 Interacts with SEL1L, a Cellular Factor That Governs Endoplasmic Reticulum-Associated Degradation of the Viral Envelope Glycoprotein gO. J. Virol..

[51] Schleiss M.R., McAllister S., Armien A.G., Hernandez-Alvarado N., Fernandez-Alarcon C., Zabeli J.C., Ramaraj T., Crow J.A., McVoy M.A. (2014). Molecular and biological characterization of a new isolate of guinea pig cytomegalovirus. Viruses.

[52] Dargan D.J., Douglas E., Cunningham C., Jamieson F., Stanton R.J., Baluchova K., McSharry B.P., Tomasec P., Emery V.C., Percivalle E. (2010). Sequential mutations associated with adaptation of human cytomegalovirus to growth in cell culture. J. Gen. Virol..

[53] Cardin R., Bravo F., Wang M., Bernstein D.I. Characterization of New Guinea Pig Cytomegalovirus Isolates: Differences in Tissue Tropism, Transplacental Transmission, and Cochlear Infection. Proceedings of the Combined meetings of 4th Congenital Cytomegalovirus Conference and 14th International CMV/Betaherpesvirus Workshop.

[54] Smith L.M., McWhorter A.R., Masters L.L., Shellam G.R., Redwood A.J. (2008). Laboratory strains of murine cytomegalovirus are genetically similar to but phenotypically distinct from wild strains of virus. J. Virol..

[55] Britt W.J., Vugler L., Butfiloski E.J., Stephens E.B. (1990). Cell surface expression of human cytomegalovirus (HCMV) gp55-116 (gB): Use of HCMV-recombinant vaccinia virus-infected cells in analysis of the human neutralizing antibody response. J. Virol..

[56] Marshall G.S., Rabalais G.P., Stout G.G., Waldeyer S.L. (1992). Antibodies to recombinant-derived glycoprotein B after natural human cytomegalovirus infection correlate with neutralizing activity. J. Infect. Dis..

[57] Pass R.F. (2009). Development and evidence for efficacy of CMV glycoprotein B vaccine with MF59 adjuvant. J. Clin. Virol..

[58] Cui X., Meza B.P., Adler S.P., McVoy M.A. (2008). Cytomegalovirus vaccines fail to induce epithelial entry neutralizing antibodies comparable to natural infection. Vaccine.

[59] McGregor A., Liu F., Schleiss M.R. (2004). Identification of essential and non-essential genes of the guinea pig cytomegalovirus (GPCMV) genome via transposome mutagenesis of an infectious BAC clone. Virus Res..

[60] Yue Y., Zhou S.S., Barry P.A. (2003). Antibody responses to rhesus cytomegalovirus glycoprotein B in naturally infected rhesus macaques. J. Gen. Virol..

[61] Ryckman B.J., Chase M.C., Johnson D.C. (2008). HCMV gH/gL/UL128-131 interferes with virus entry into epithelial cells: Evidence for cell type-specific receptors. Proc. Natl. Acad. Sci. USA.

[62] Schafer H., Klippert K., Meuer P., Borsdorf B., Kiderlen A.F., Burger R. (2007). Biologic activity of guinea pig IFN-gamma in vitro. J. Interferon Cytokine Res..

[63] McGregor A., Choi K.Y., Schleiss M.R. (2011). Guinea pig cytomegalovirus GP84 is a functional homolog of the human cytomegalovirus (HCMV) UL84 gene that can complement for the loss of UL84 in a chimeric HCMV. Virology.

