# Supplementary material for: Convalescent Immunity to Guinea Pig Cytomegalovirus Induces Limited Cross Strain Protection against Re-Infection but High-Level Protection against Congenital Disease
Source: Int J Mol Sci. 2020 Aug 20;21(17):5997. doi: 10.3390/ijms21175997 (PMC7504201; doi:10.3390/ijms21175997)

## Slide 1
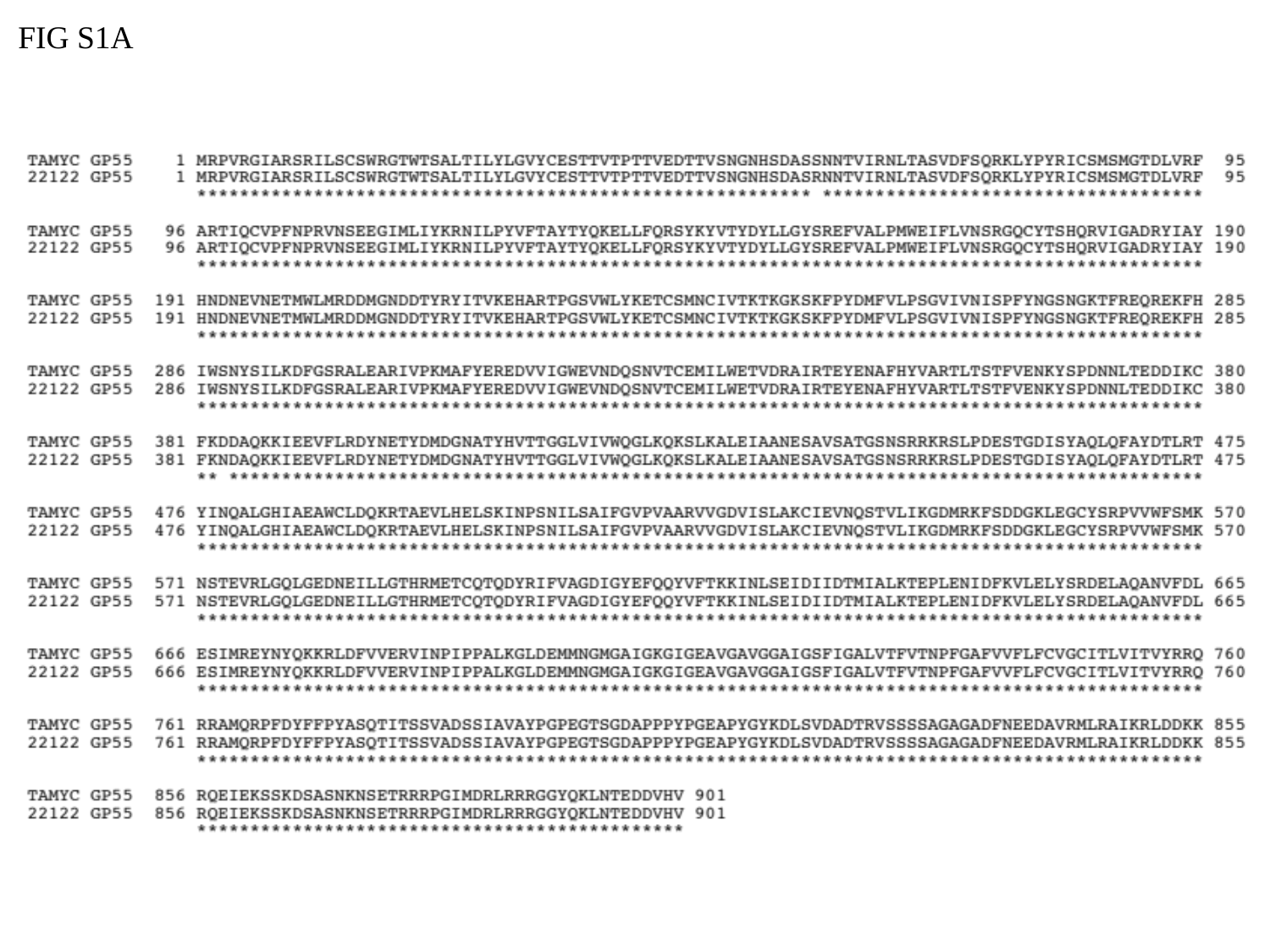

FIG S1A

## Slide 2
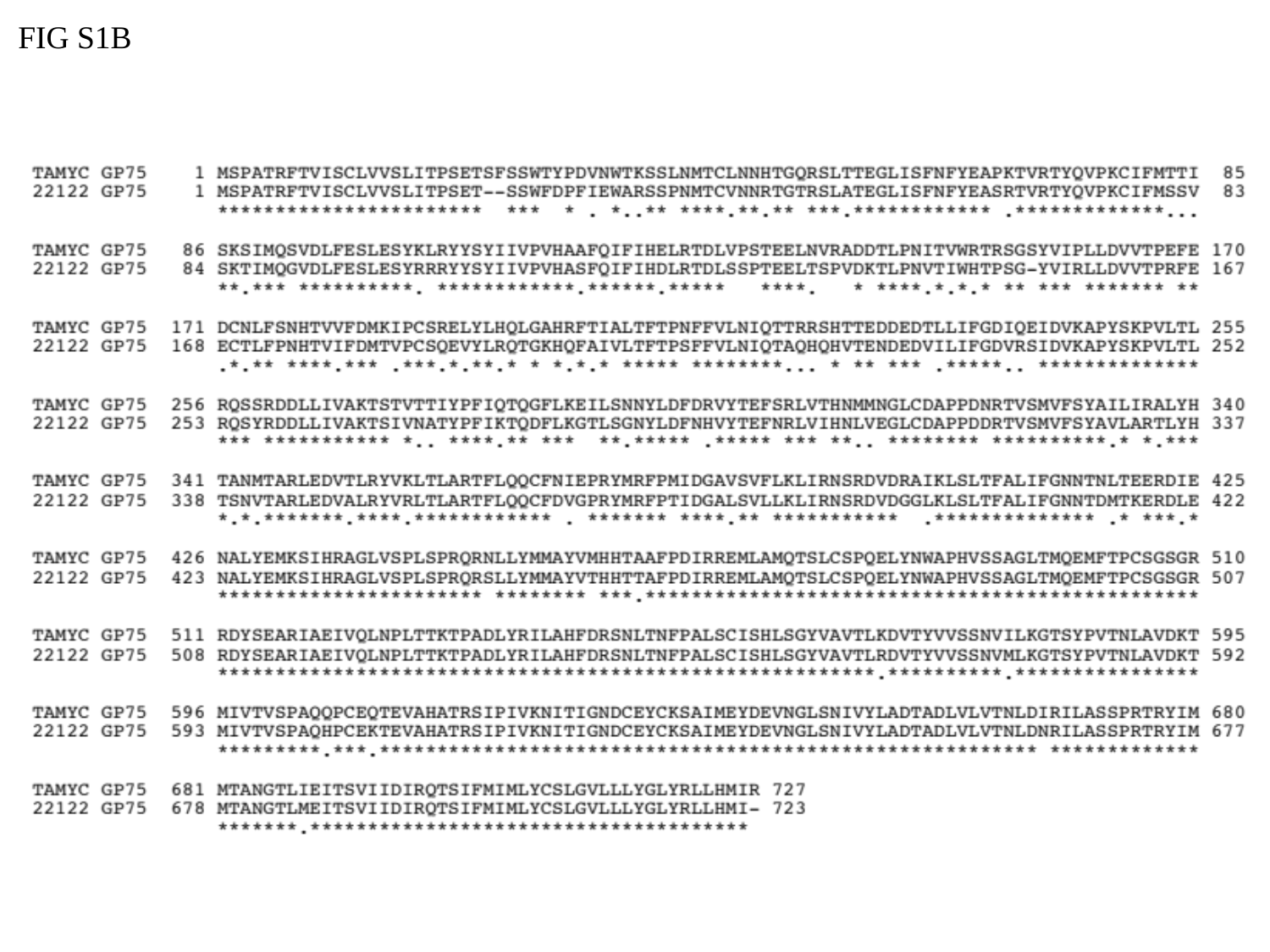

FIG S1B

## Slide 3
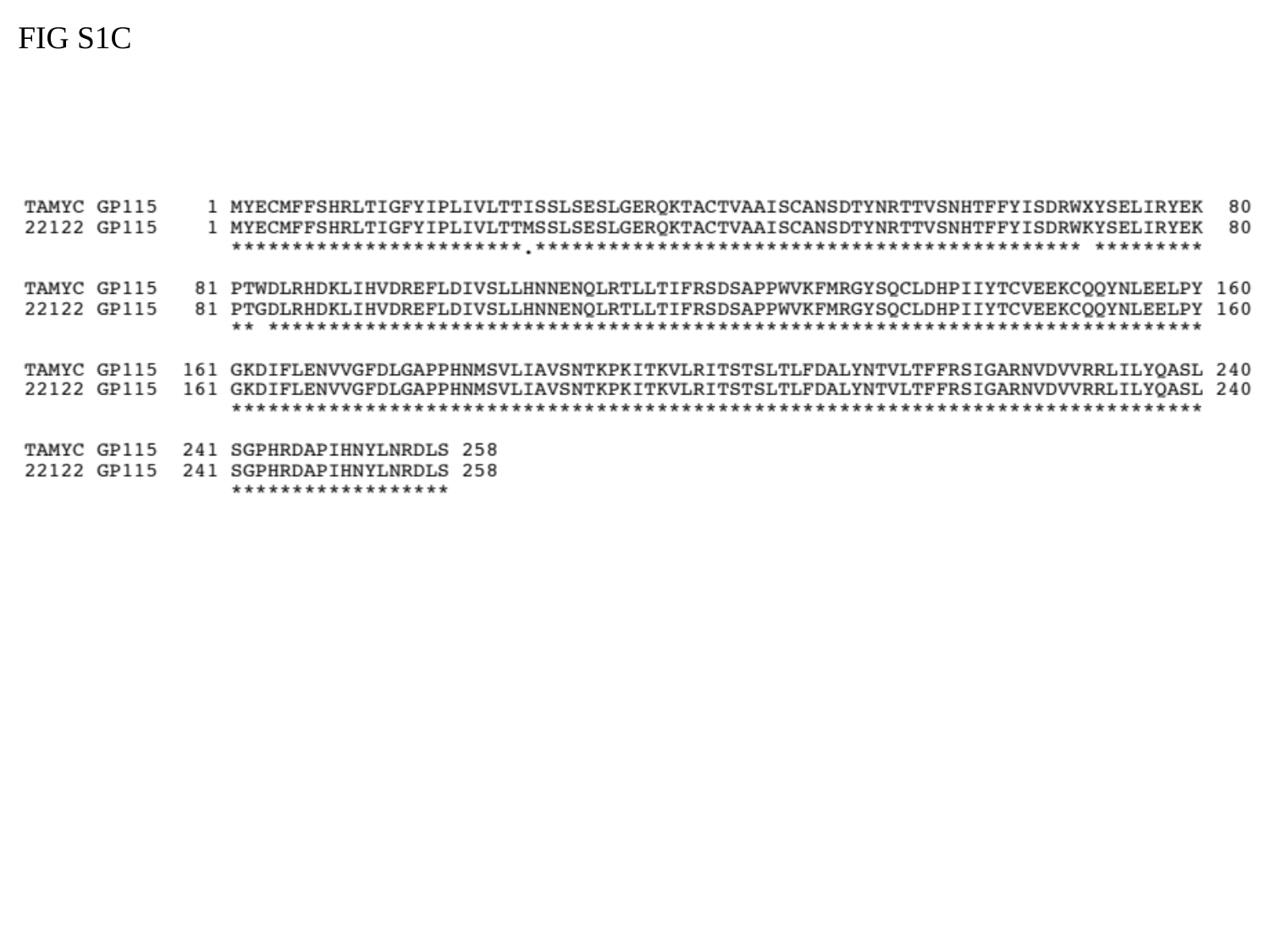

FIG S1C

## Slide 4
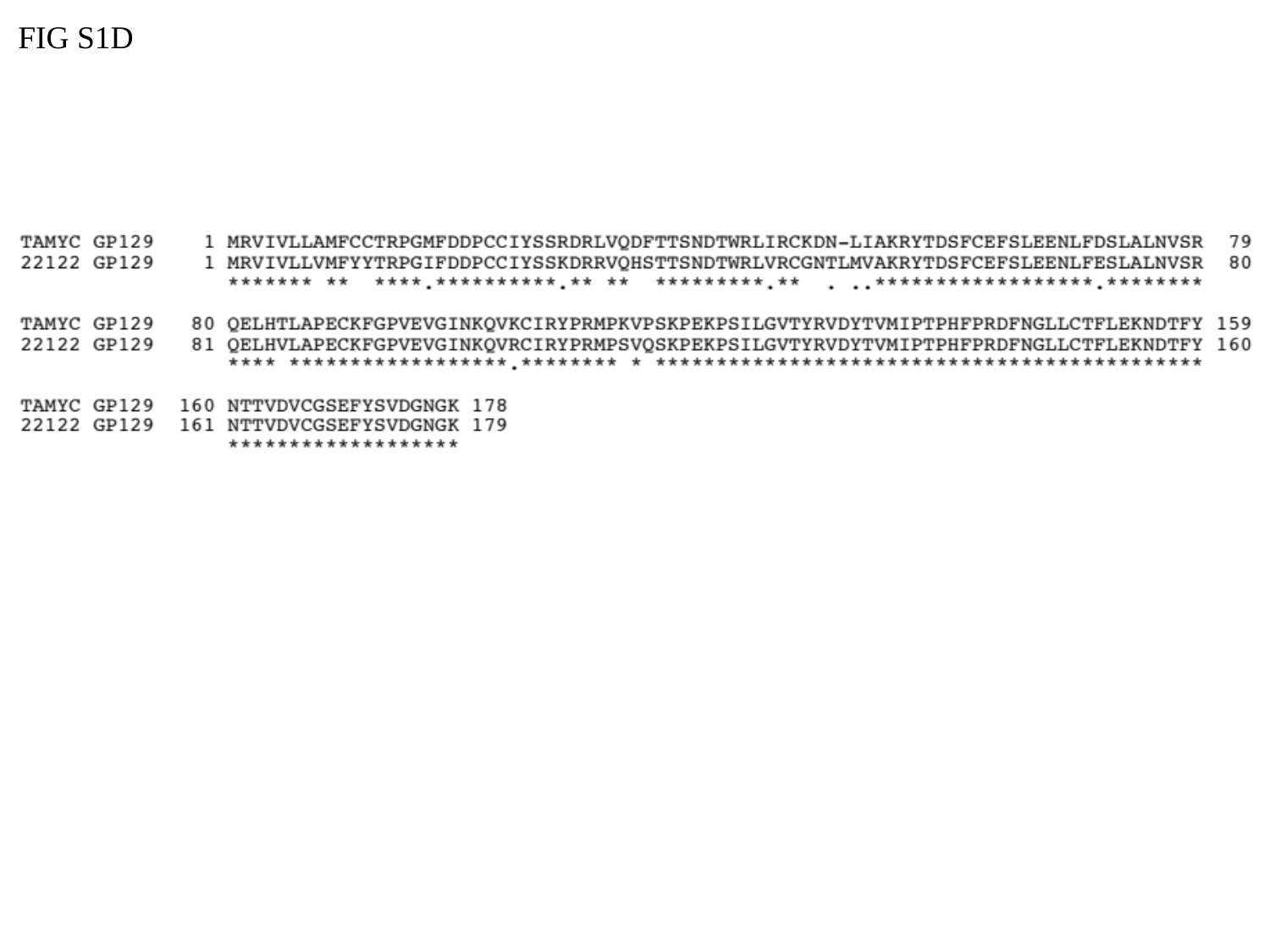

FIG S1D

## Slide 5
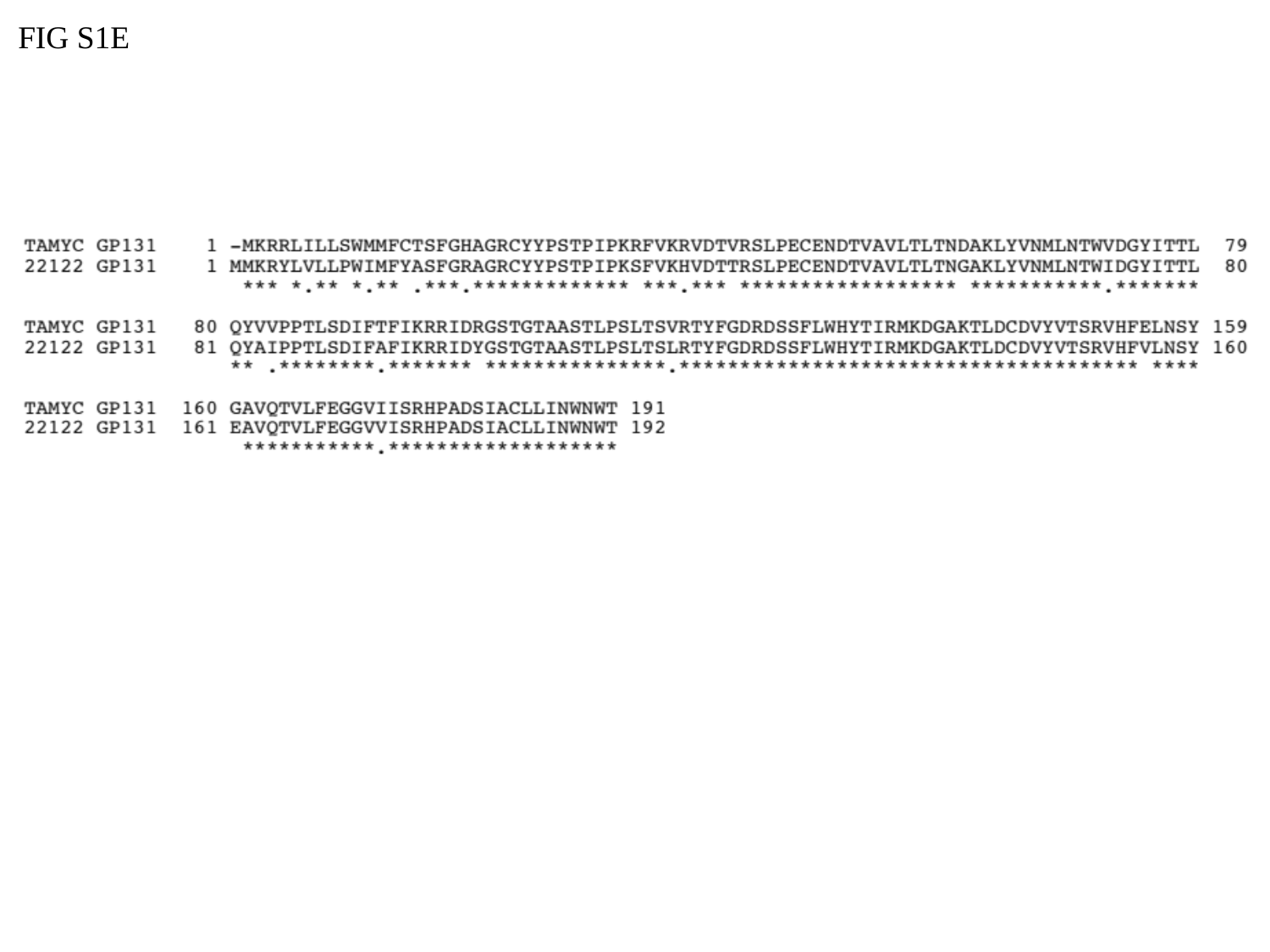

FIG S1E

## Slide 6
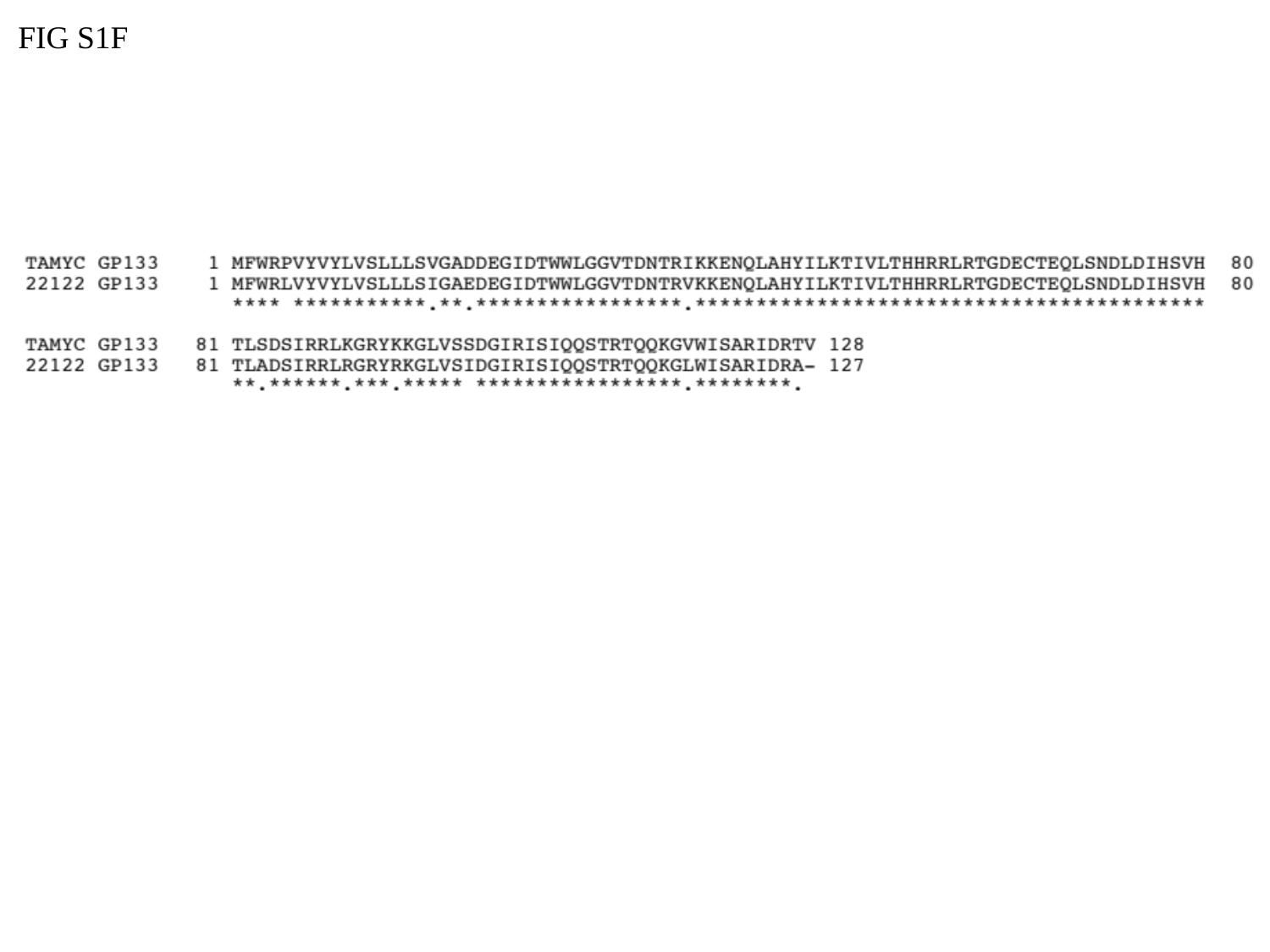

FIG S1F

## Slide 7
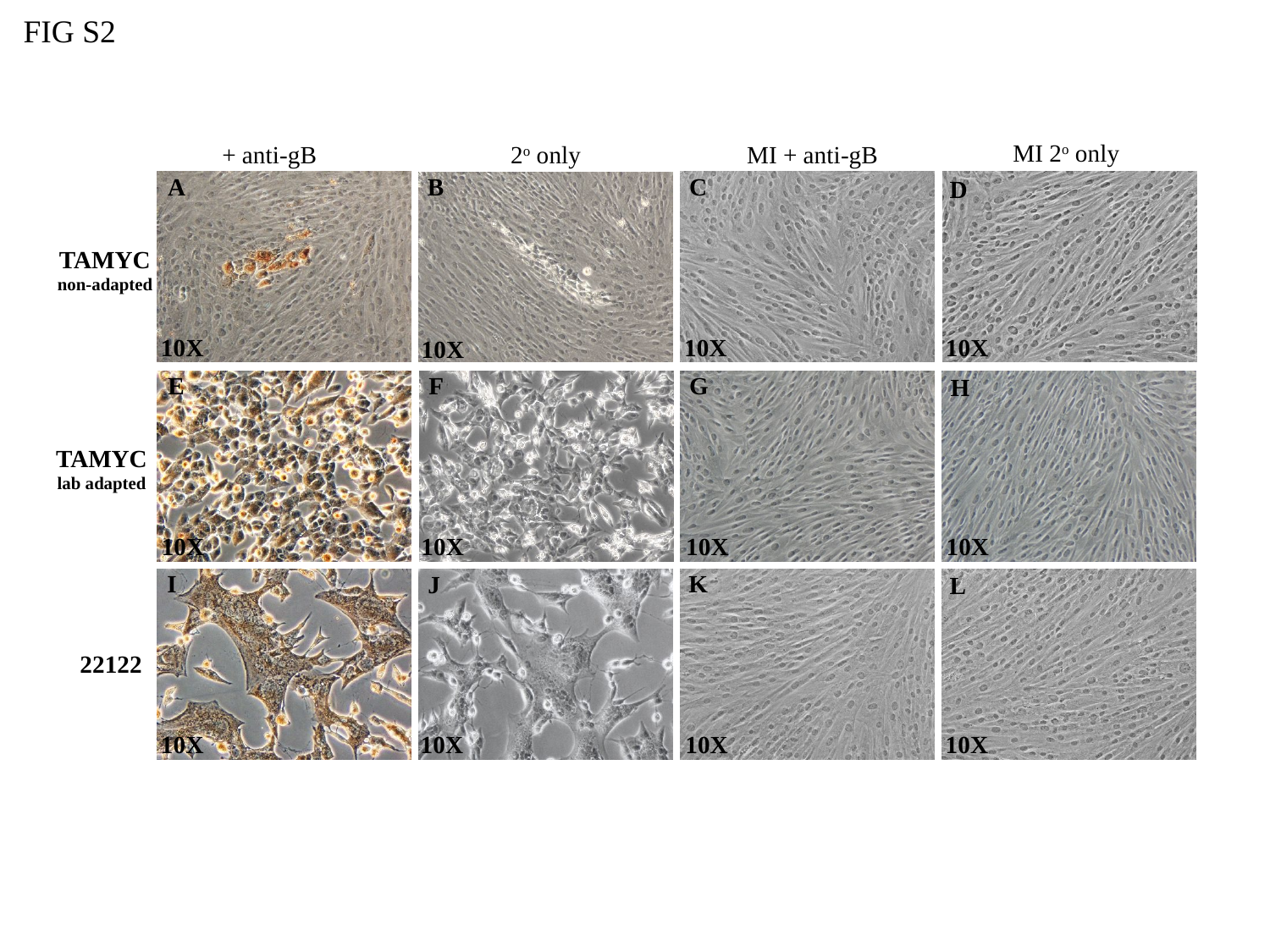

FIG S2
MI 2o only
2o only
MI + anti-gB
+ anti-gB
B
C
A
D
10X
10X
10X
10X
TAMYC
non-adapted
E
G
F
H
TAMYC
lab adapted
10X
10X
10X
10X
I
K
J
L
22122
10X
10X
10X
10X

## Slide 8
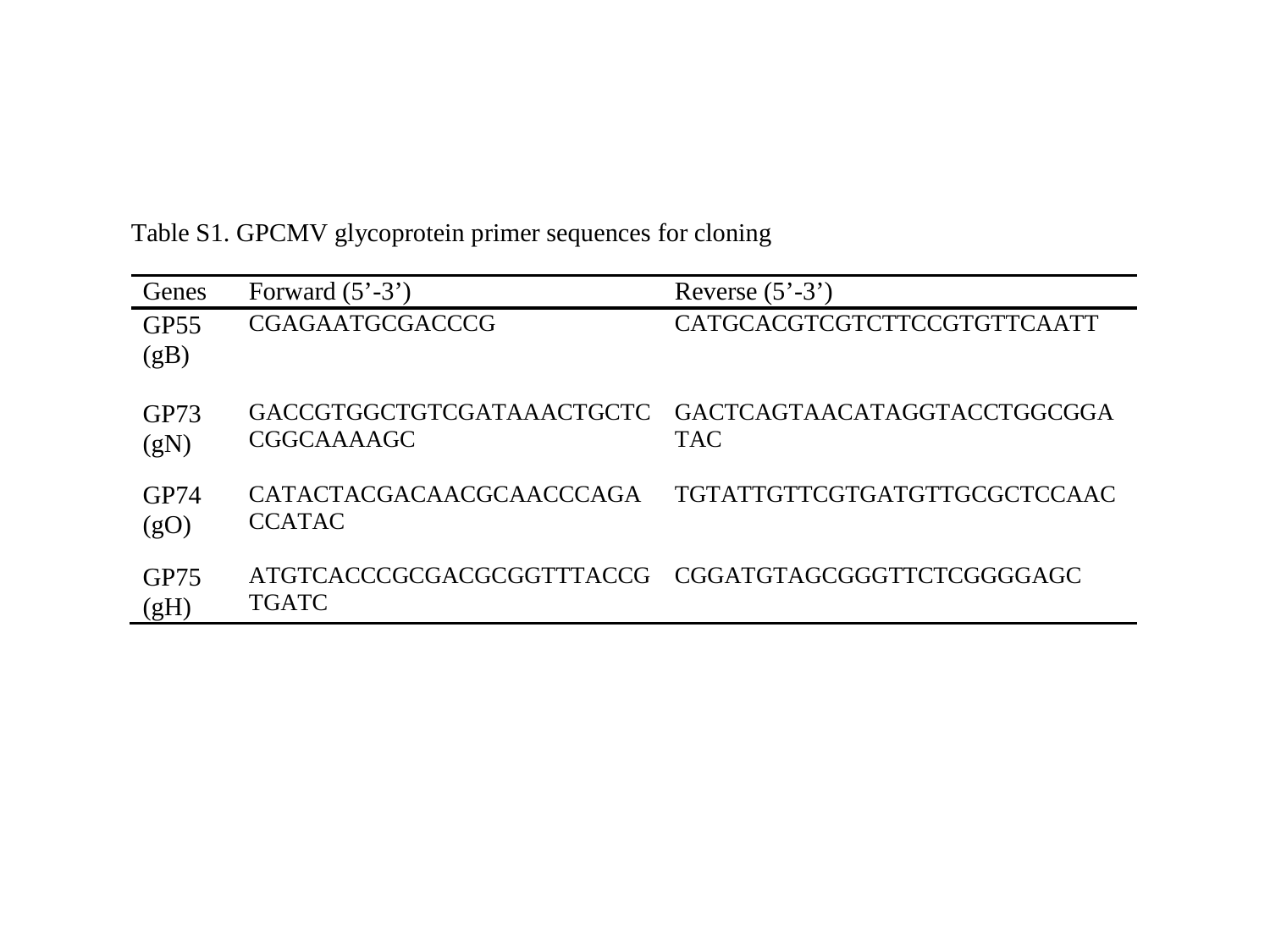

Supplement: Supplementary file 1 [file ijms-21-05997-s001.pptx]
